# Empty Sella Syndrome

**DOI:** 10.51894/001c.165677

**Published:** 2026-08-06

**Authors:** Haoran Li

**Affiliations:** 1 McLaren Greater Lansing

## 92

### Background

Empty Sella Syndrome is a radiologic finding in which CT or MRI imaging reveals a small or flattened pituitary gland within the sella turcica. Empty Sella Syndrome occurs in two forms: primary, which is idiopathic, and secondary, which is associated with conditions such as idiopathic intracranial hypertension, pituitary surgery, radiation therapy, trauma, or infarction.

### Case Presentation

A 38-year-old woman presented to the clinic for emergency department follow-up. She reported visiting the ED a few months earlier for neck pain after a motor vehicle accident. During her evaluation, a CT scan of the head and neck revealed an incidental finding of an empty pituitary fossa, with no evidence of acute cervical or skull fracture or intracranial bleeding. She received pain management and was discharged the same day with instructions to follow up with her primary care provider.

### Conclusion

This case highlights the uncommon radiologic finding known as Empty Sella Syndrome. Many patients with this condition have a flattened pituitary gland, which may not be visible on CT imaging and can be misinterpreted as an “empty pituitary fossa.” MRI can be used to confirm the presence of the pituitary gland. In patients without signs of endocrine insufficiency, expectant management with periodic surveillance is appropriate.

